# Cardiac vagal activity is associated with insulin metabolism in heart failure: Results from the Myovasc study

**DOI:** 10.1186/s12933-025-03040-9

**Published:** 2026-01-08

**Authors:** Noémie Bélanger, Silav Zeid, David Velmeden, Andreas Schulz, Thomas Koeck, Felix Rausch, Benedikt Fooß, Fawad Kazemi-Asrar, Karl J. Lackner, Tommaso Gori, Tommaso Münzel, Jürgen H. Prochaska, Perikles Simon, Philipp S. Wild

**Affiliations:** 1 Preventive Cardiology and Preventive Medicine, Department of Cardiology and Clinical Epidemiology and Systems Medicine, Center for Thrombosis and Hemostasis, University Medical Center of the Johannes Gutenberg-University Mainz, Langenbeckstr. 1, 55131 Mainz, Germany; 2 German Center for Cardiovascular Research (DZHK), partner site Rhine-Main, Mainz, Germany; 3 Institute for Clinical Chemistry and Laboratory Medicine, University Medical Center of the Johannes Gutenberg University Mainz, Mainz, Germany; 4 Department of Cardiology – Cardiology I, University Medical Center of the Johannes Gutenberg University Mainz, Mainz, Germany; 5 Clinical Epidemiology and Systems Medicine, Center for Thrombosis and Hemostasis (CTH), University Medical Center of the Johannes Gutenberg University, Mainz, Germany; 6 Boehringer Ingelheim International GmbH, Ingelheim, Germany; 7 Sports Medicine and Disease Prevention, Department of Sports Medicine, Prevention and Rehabilitation, Johannes Gutenberg-University Mainz, Mainz, Germany; 8 Systems Medicine, Institute of Molecular Biology (IMB), Mainz, Germany

**Keywords:** Insulin resistance, Hyperglycemia, C-peptide, Heart failure, Heart rate recovery, Cardiac autonomic dysfunction, Parasympathetic reactivation

## Abstract

**Background:**

Cardiac autonomic dysfunction plays a pivotal role in the heart failure syndrome. Metabolic dysregulation affects both autonomic function and heart failure, but these relationships remain incompletely understood. This study aimed at investigating the role of glucose and insulin metabolism for parasympathetic reactivation.

**Methods:**

Data from the MyoVasc study (NCT04064450), a prospective heart failure cohort study, were analyzed. Participants underwent a highly standardized 5-hour examination, including venous blood sampling. To assess the impact of glucose and insulin metabolism (HbA_1c_, HOMA-IR, and C-peptide) on parasympathetic reactivation as reflected by heart rate recovery 60 s (HRR_60_) after cardiopulmonary exercise testing, multivariable linear regression models with adjustment for sex, age, clinical profile (cardiovascular risk factors and comorbidities) and medication were calculated in cross-sectional and longitudinal settings. Additional adjustment for complementary glucose or insulin status was performed to assess the dependency of each other. Analyses were carried out in symptomatic heart failure and across the spectrum of glucose metabolism dysfunction.

**Results:**

The analysis sample included 1,588 individuals (median age 64.0 years [IQR 55.0;72.0]; 33% women) in a fasting state. Symptomatic heart failure was present in 43.7% of the subjects. Median HRR_60_ was 21.0 beats per minute (IQR 14.0;28.0). In multivariable regression analysis with adjustment for age, sex, clinical profile, and medication, both HbA_1c_ ($\:\widehat{{\upbeta\:}}$_per SD_ −0.074, 95% CI [− 0.122;−0.026], *P* = 0.003) and HOMA-IR ($\:\widehat{{\upbeta\:}}$_per SD_ −0.113 [− 0.165;−0.062], *P* < 0.0001) predicted HRR_60_. Additional adjustment for both glucose and insulin status, respectively, demonstrated that HOMA-IR ($\:\widehat{{\upbeta\:}}$_per SD_ −0.097 [− 0.155;−0.040], *P* < 0.0001), but not HbA_1c_ ($\:\widehat{{\upbeta\:}}$_per SD_ −0.030 [− 0.084;0.025], *P* = 0.28), was independently related to HRR_60_. This finding was confirmed in subgroups with heart failure and type 2 diabetes. In all analyses, C-peptide was related to HRR_60_ independently of HbA_1c_ with higher effect estimates than HOMA-IR ($\:\widehat{{\upbeta\:}}$_per SD_ −0.171 [− 0.225;−0.117], *P* < 0.0001). Finally, higher HbA_1c_ ($\:\widehat{{\upbeta\:}}$_per SD_ −0.094, [− 0.171;−0.017], *P* = 0.017) and C-peptide ($\:\widehat{{\upbeta\:}}$_per SD_ −0.076, [− 0.159;0.007], *P* = 0.075) were more strongly associated with a lower HRR_60_ after two years of follow-up.

**Conclusions:**

This study demonstrates the relevance of insulin status for vagal activity of cardiac autonomic function, particularly in heart failure. The pathophysiological implications underlying the relationship between insulin status and parasympathetic activity merit further mechanistic exploration.

**Supplementary Information:**

The online version contains supplementary material available at 10.1186/s12933-025-03040-9.

## Research Insights

What is currently known about this subject?

- Both cardiac autonomic dysfunction and metabolic dysregulation play critical roles in the development of heart failure.
- Heart rate recovery after 60 seconds (HRR_60_), indicative of parasympathetic reactivation after exercise, appears as an interesting marker to assess metabolic health in heart failure, with known associations to both type 2 diabetes mellitus and heart failure.

What is the key research question?

How do insulin and glucose metabolism contribute to vagal activity in cardiac autonomic function in the context of heart failure?

What is new?

- Impaired glucose metabolism was associated with reduced parasympathetic reactivation as measured by HRR_60_ regardless of clinical profile and medication, but not independently of insulin status.
- Insulin resistance, as represented by HOMA-IR, was related to reduced HRR_60_ independently of clinical profile, medication and also glycemic status.
- C-peptide was related with HRR_60_ independently of glycemic status, but with higher effect estimates than for HOMA-IR cross-sectionally and longitudinally.

How might this study influence clinical practice?

This study demonstrates the relevance of insulin status for vagal activity of cardiac autonomic function, including in individuals with heart failure. This might create opportunities for interventions in individuals with autonomic dysfunction.

## Introduction

Heart failure is a clinical syndrome with complex pathophysiology, in which neuroendocrine systems and metabolic states, such as type 2 diabetes mellitus, play an important role [1]. Cardiac autonomic dysfunction, characterized by an overactivity of the sympathetic nervous system and inhibition of the parasympathetic nervous system, is also a major contributor to the development and progression of heart failure [1, 2]. The release of catecholamines that accompanies the overactivation of the sympathetic nervous system has positive inotropic and chronotropic effects on the heart [1], with constantly elevated levels of catecholamines causing cardiac hypertrophy and inflammation [3, 4]. 

Moreover, the normal drop in heart rate post-exercise is often markedly diminished in heart failure, as subjects exhibit reduced vagal tone, which has been associated with poor outcomes [5, 6]. Heart rate recovery after 60 seconds (HRR_60_) is considered a non-invasive and easily interpretable marker of parasympathetic cardiac autonomic dysfunction [7]. The sympathetic nervous system predominates during exercise, but cardiac vagal reactivation mainly defines the first minute after cessation of peak exercise [8]. Thus, a delayed or blunted HRR_60_ suggests impaired vagal reactivation and autonomic imbalance. It was also shown to be a reliable predictor of mortality and the onset of type 2 diabetes mellitus [8–10]. 

Autonomic dysfunction has been pointed out as both a cause and a complication of type 2 diabetes, with hyperglycemia being a considerable culprit [11]. For instance, type 2 diabetes-related chronic hyperglycemia is a major factor in the development of cardiac autonomic neuropathy, in part due to oxidative stress and alterations to neuronal metabolism [12]. Moreover, hyperinsulinemia is known to impact autonomic function by elevating sympathetic nervous system activity, which, apart from being an essential feature of heart failure, also predicts long-term cardiometabolic risk factors, such as obesity and arterial hypertension [13, 14]. While glucose and insulin metabolism are known to affect autonomic function, most literature focuses on type 2 diabetes as a whole, neglecting the presumably independent effects of insulin resistance and hyperglycemia [15]. This distinction is of relevance as these conditions do not necessarily occur concomitantly, especially in early disease stages or prediabetes. This is particularly important as clinical type 2 diabetes is often diagnosed much later than its pathophysiological onset [16]. Very few studies from the early 2000s with limited sample size suggest a greater impact of glucose and insulin metabolism on cardiac autonomic function [17, 18], but this has not been investigated in the context of heart failure, despite the critical roles played by autonomic dysfunction and metabolic dysregulation in its development [19]. 

Given the high risk of type 2 diabetic patients to develop heart failure and the relevance of autonomic dysfunction in both conditions, their complex interactions warrant further investigation [11, 20]. This project aimed to better understand the effect of glucose and insulin metabolism on autonomic dysfunction in heart failure. Specifically, it assessed the separate and combined effects of insulin resistance and hyperglycemia on HRR_60_ (1) in heart failure and its phenotypes and across the type 2 diabetes spectrum and (2) at different time points to investigate if insulin and glucose metabolism were associated with a deterioration of cardiac vagal status over time.

## Methods

### Study design and cohort

The MyoVasc study (NCT04064450, *N* = 3,289) is a large prospective, single-center cohort of individuals with heart failure established in 2013 with structured follow-up [21]. The study is conducted at the University Medical Center of the Johannes Gutenberg University Mainz, in Mid-Western Germany, where individuals undergo a highly standardized five-hour examination every two years in a dedicated study center. Participants also receive every year a detailed computer-assisted telephone interview to record clinical status, medication intake, and adverse events [21]. All study participants provided written consent prior to enrollment. All investigations were assessed by the relevant ethics committees before and over the course of the study (reference number 837.319.12 [8420-F]). The data safety commissioner also determined compliance with the law before study initiation. Protocols and study staff followed the Declaration of Helsinki and the recommendations of Good Clinical Practice and Good Epidemiological Practice throughout all study procedures.

Participants were categorized in heart failure stages following the Universal definition of heart failure (at risk for heart failure [Stage A], pre-heart failure [Stage B], symptomatic heart failure [Stage C] and advanced heart failure [Stage D]) [22]. Participants without heart failure or at risk for heart failure were recruited by a sample from the general population. Subjects undergo extensive biobanking and phenotyping every two years, which includes, with relevance for the present work, NT-proBNP [pg/ml], HbA_1c_ [%], glucose [mg/dL], insulin [pmol/L], C-peptide (ng/mL), proinsulin [pmol/L], and other laboratory markers in a fasted state as well as a 12-lead resting electrocardiogram, 2D and 3D transthoracic echocardiography, and 24-hour Holter ECG. Left ventricular ejection fraction (LVEF) was derived using Simpson’s method in apical four-chamber view. All data were quality controlled for accuracy by a data management unit.

The five-hour investigation conducted every two years includes cardiopulmonary exercise testing (CPET), performed on a VIAsprint cycle ergometer (Ergoline, Bitz, Germany) using either the WHO-25 or WHO-50 protocol, adapted to the participant’s fitness level. These stepwise protocols involve workload progressive increments every three minutes and are selected based on individual functional capacity. Testing continued until the participant reached volitional exhaustion or clinical criteria required termination. Electrocardiographic data were recorded using the GE KISS system in combination with GE CardioSoft (v6.71). Ventilatory parameters were collected via the Jaeger MS-CPX system, and ventilatory thresholds were determined using modified Wasserman curves implemented in JLab software (v5.72.1.77). Other absolute criteria for CPET termination and more details on the CPET protocols can be found in the Supplementary Methods. CPET was performed in an overnight fasted state. Sixty seconds after completion of CPET (and cessation of peak strain), HRR was measured in a sitting position on the cycle ergometer. All equipment was calibrated before the examination.

Individuals with an available HRR_60_ were included in this study. Exclusion criteria comprised individuals (i) with a pacemaker, atrial fibrillation or atrial flutter based on a resting ECG performed on the day of the examination, or who had undergone heart transplantation; (ii) with type 1 diabetes mellitus, and (iii) fasting for less than five hours.

### Statistical analyses

Descriptive characteristics of the whole sample were reported in absolute and relative frequencies for dichotomous variables and for continuous variables by their median (with interquartile range [IQR]). HbA_1c_ and HOMA-IR, established markers of glucose and insulin status, were analyzed as continuous measures at baseline. Multiple linear regression models were used to compare the impact of HbA_1c_ and HOMA-IR on log-transformed HRR_60_ in the overall sample as well as within clinically relevant subgroups defined by heart failure stages and phenotypes, and across the type 2 diabetes mellitus spectrum (i.e. euglycemia, prediabetes, and type 2 diabetes). Within the type 2 diabetes subgroup, individuals managing their condition exclusively through dietary interventions were excluded. For the analysis, controls and heart failure stage A were grouped together, as well as heart failure stages C and D. Heart failure phenotypes were defined according to LVEF in the heart failure sample (stages C and D). Heart failure with preserved ejection fraction (HFpEF) was defined as LVEF > 50% and heart failure with reduced ejection fraction (HFrEF) as LVEF ≤ 50%. As all analyses were exploratory, no correction was applied for multiple testing.

Estimates were standardized per standard deviation (SD), in the case of numerical predictors such as the glucose and insulin markers, to allow for direct comparison and were presented with a 95% confidence interval (CI). Models were adjusted for possible confounders: age, sex, and the cardiovascular risk factors arterial hypertension, dyslipidemia, family history of myocardial infarction or stroke, obesity (BMI ≥ 30 kg/m^2^) or BMI as a continuous variable, and smoking in the past seven years, and the comorbidities atrial fibrillation based on anamnesis, chronic kidney disease, chronic obstructive pulmonary disease, coronary artery disease, history of cancer, history of myocardial infarction, history of stroke, peripheral artery disease, and venous thromboembolism, as well as medication intake. Heavy alcohol consumption, defined as 24 g/day for men and 12 g/day for women, was also accounted for. A final adjustment for complementary glucose or insulin status was performed to analyze the independent relationship between insulin or glucose status and HRR_60_. Medication with an effect on HRR_60_ was assessed with an unbiased approach by an individual medication score, as previously described [10]. The score was derived from an unadjusted linear regression model based on the estimated effects of commonly used drug classes, including agents for cardiovascular, metabolic, neurological, and respiratory conditions that showed significant associations with HRR_60_ in the overall cohort.

Multiple linear regression analysis was performed to investigate the impact of antidiabetic medication on HRR_60_. Metformin and insulin were analyzed in combination and independently. Confounders comprised clinical profile (cardiovascular risk factors and comorbidities), medications other than antidiabetic agents, and additionally included duration of type 2 diabetes (in years), NT-pro-BNP and NYHA functional class to account for a worse clinical status in those taking insulin and analogues.

Final analyses exploring longitudinal data were similarly conducted in the whole sample and in symptomatic heart failure. The predictive value of HbA_1c_ and HOMA-IR for the HRR_60_ after 2 years of follow-up was investigated with adjustment for baseline values of HRR_60_ to assess a change in HRR_60_ over time, and additionally for cardiovascular risk factors, comorbidities, and medication intake. To confirm the results yielded with HOMA-IR, all aforementioned analyses (cross-sectional and sequential) were also performed with insulin and C-peptide as predictors. Proinsulin was also investigated in the whole analysis sample and in the symptomatic HF subsample.

Furthermore, to ensure that maximal exertion, and thus peak heart rate, was achieved during exercise, a sensitivity cross-sectional analysis was performed in a subsample of individuals who achieved a respiratory exchange ratio (RER) > 1.0 [23]. Models were adjusted for the same adjustments previously mentioned, including complementary insulin or glucose status. The CPET protocol, as a confounder for impaired functional capacity, was also accounted for.

Exploratory analyses with heart rate variability markers derived from 24-hour Holter ECG (standard deviation of all NN intervals [SDNN), low-frequency to high-frequency ratio [LF/HF ratio], and root mean square of successive differences [RMSSD]) were also performed.

*P*-values were used as continuous measures of evidence given the explorative nature of this study. All analyses were performed using the statistical software R, version 4.1.0.

## Results

A total of *N* = 1,588 individuals (median age 64.0 years [IQR 55.0; 72.0]; 33% female sex) from the MyoVasc cohort were included in the analysis sample (Supplementary Fig. 1). Clinically established symptomatic heart failure was present in 43.7% of the analysis sample. A total of 19.0% of participants had type 2 diabetes. Median HRR_60_ was 21.0 bpm (IQR 14.0; 28.0). A total of 46.5% of the individuals achieved a RER > 1.0. The CPET protocol starting at 25 W was carried out by 9.1% (*n* = 145) of participants. The clinical characteristics of the overall sample are displayed in Table 1, and additional information including medication intake is provided in Supplementary Table 1. Clinical characteristics according to heart failure stage are displayed in Supplementary Table 2. A sensitivity analysis found no major differences between five and eight hours of fasting in relation to insulin and glucose metabolism markers (Supplementary Table 3). Therefore, all individuals were included in further analyses.

**Table 1:** Baseline characteristics of the analysis sample

Characteristics	
*n*	1,588
Age in years	64.0 (55.0 − 72.0)
Sex, % women (n)	33.0 (524)
BMI in kg/m²	27.4 (24.8 − 30.6)
HRR_60_ at baseline in bpm	21.0 (14.0 − 28.0)
*Heart failure stages/Universal definition of heart failure, % (n)*	
No heart failure (Stage 0/A)	22.9 (363)
Pre-heart failure (Stage B)	33.4 (531)
Symptomatic heart failure (Stage C/D)	43.7 (694)
*Glucose and insulin-related characteristics*	
Euglycemia, % (n)	67.5 (1,072)
Prediabetes, % (n)	13.5 (214)
Type 2 diabetes mellitus, % (n)	19.0 (302)
HbA_1c_ in %	5.70 (5.40 − 6.10)
HOMA-IR	1.62 (1.07 − 2.55)
C-peptide in ng/mL	1.82 (1.34 − 2.54)
Proinsulin in pmol/l	3.98 (2.57 − 6.82)
Antidiabetic medication, % (n)	14.3 (227)
Insulins and analogues, % (n)	4.7 (74)
Biguanides, % (n)	8.0 (127)
*Cardiovascular risk factors, % (n)*	
Arterial hypertension	68.3 (1085)
Dyslipidemia	69.0 (1095)
Family history of myocardial infarction/stroke	22.5 (357)
Obesity	27.8 (441)
Smoking in the last 7 years	23.0 (365)
*Comorbidities, % (n)*	
Atrial fibrillation based on anamnesis	15.6 (248)
Chronic kidney disease, eGFR < 60 ml/min/1.73 m^2^	12.3 (194)
Chronic obstructive pulmonary disease	20.1 (319)
Coronary artery disease	37.0 (587)
History of cancer	14.9 (237)
History of myocardial infarction	24.3 (386)
History of stroke	7.2 (115)
Peripheral artery disease	4.9 (78)
Venous thromboembolism	7.3 (115)

All continuous variables are presented as the median with the interquartile range. BMI, body mass index; HRR_60_, heart rate recovery at 60 s post-exercise; bpm, beats per minute; eGFR, estimated glomerular filtration rate. Participants considered to have atrial fibrillation based on anamnesis were in sinus rhythm during examination.

### Comparison of the relation between insulin and glucose status with HRR_60_

Both HbA_1c_ and HOMA-IR were predictors of HRR_60_ (respectively, $\:\widehat{{\upbeta\:}}$ estimate _per SD_ −0.074, 95% CI [− 0.122; −0.026], *P* = 0.003 and $\:\widehat{{\upbeta\:}}$_SD_ −0.113 [− 0.165; −0.062], *P* < 0.001), independent of age, sex, clinical profile and medication intake. Similar results were obtained when substituting adjustment for obesity by adjustment for BMI as a continuous variable (HbA_1c_: $\:\widehat{{\upbeta\:}}$_SD_ −0.06 [− 0.11; −0.01], *P* = 0.016 and HOMA-IR: −0.10 [− 0.15; 0.04], *P* = 0.00034). After additional adjustment for respective complementary glucose and insulin metabolism, HOMA-IR remained independently related to HRR_60_ as opposed to HbA_1c_ (Table 2). Medication related to HRR_60_ is described in the Supplementary Results.

**Table 2:** Relationship between glucose and insulin status and heart rate recovery after 60 s

	Adjusted for sex and age		Additionally adjusted for cardiovascular risk factors, comorbidities, and medication		Additionally adjusted for complementary glucose/insulin status^Φ^	
	$\:\widehat{{\upbeta\:}}$-estimate per SD [95% CI]	*p*-value	$\:\widehat{{\upbeta\:}}$-estimate per SD [95% CI]	*p*-value	$\:\widehat{{\upbeta\:}}$-estimate per SD [95% CI]	*p*-value
HbA_1c_	−0.220 [− 0.268; −0.173]	< 0.0001	−0.074 [− 0.122; −0.026]	0.0027	−0.030 [− 0.084; 0.025]	0.28
HOMA-IR	−0.279 [− 0.327; −0.232]	< 0.0001	−0.113 [− 0.165; −0.062]	< 0.0001	−0.097 [− 0.155; −0.040]	0.0010

The $\:\widehat{{\upbeta\:}}$-estimates [per SD] presented are from multivariate regression analyses in the whole analysis sample (*n* = 1,575). HbA_1c_ and HOMA-IR are the independent variables and HRR_60_ is the dependent variable. HRR_60_ is log-transformed.

^Φ^ Complementary glucose/insulin status indicates that HbA_1c_ was adjusted for HOMA-IR and vice versa. SD, standard deviation; CI, confidence interval

*Relationship between insulin and glucose status with HRR*_*60*_ *across heart failure stages and across the spectrum of glucose disturbances*.

**Fig. 1:**
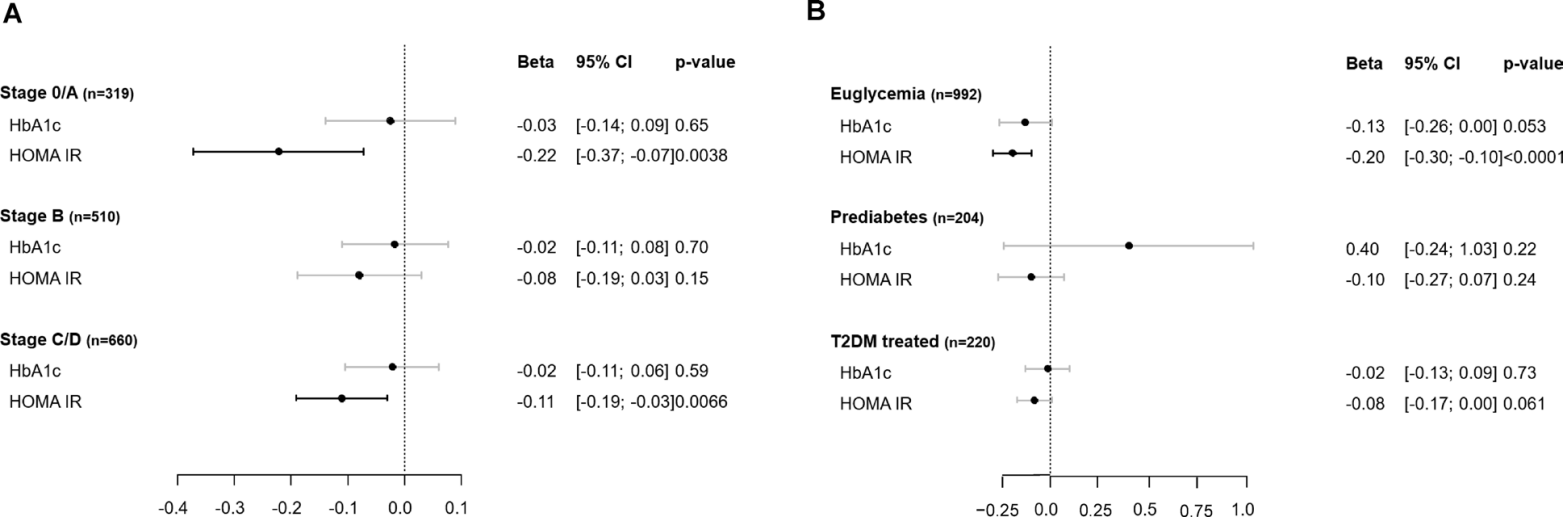
The relationship between glucose and insulin status and HRR_60_ stratified by heart failure stages (**A**) and glycemic states (**B**). The $\hat{\beta }$ estimates [per standard deviation] with a 95% CI presented are from multivariate regression models with HbA1c and HOMA-IR as independent variables and HRR_60_ as dependent variable. HRR_60_ was log-transformed. Results are presented according to heart failure stages in panel A and according to glycemic state in panel B. Type 2 diabetic individuals with only dietary treatment were excluded from the analysis (n = 75). The models are adjusted for sex, age, cardiovascular risk factors, comorbidities, medication and insulin or glucose status. HbA_1c_ was adjusted for HOMA-IR and vice versa. CI, confidence interval

The relationship between glucose and insulin metabolism and HRR_60_ stratified by heart failure stages is presented in Fig. 1A. A relevant contribution of HOMA-IR to HRR_60_ was observed in individuals without heart failure, independently of age, sex, clinical profile, and respective complementary glucose and insulin metabolism (HOMA-IR: $\:\widehat{{\upbeta\:}}$_SD_ −0.223 [− 0.373; −0.073], *P* = 0.004), while no relationship was observed for HbA_1c_ under the same adjustments. In pre-heart failure, the association between HOMA-IR and HRR_60_ as well as HbA1c and HRR60 was not present (Fig. 1A). In symptomatic heart failure, insulin status remained related to HRR_60_ (HOMA-IR: $\:\widehat{{\upbeta\:}}$_SD_ −0.111 [− 0.191; −0.031], *P* = 0.007), whereas no association was observed for HbA_1c_. Models with adjustment for age and sex only and models with additional adjustment for complementary glucose or insulin status are shown in Supplementary Table 4. HOMA-IR was more strongly associated with lower HRR_60_ than HbA_1c_ in both HFrEF and HFpEF, with no evidence of interaction between the heart failure phenotypes (*P* = 0.99 for HbA_1c_; *P* = 0.60 for HOMA-IR; Supplementary Table 5).

The association between HOMA-IR and HbA_1c_ and HRR_60_ across the type 2 diabetes spectrum is displayed in Fig. 1B with adjustment for sex, age, clinical profile, medication intake and insulin or glucose status. In euglycemic individuals, both HbA_1c_ and HOMA-IR predicted HRR_60_, with HOMA-IR having stronger association ($\:\widehat{{\upbeta\:}}$_SD_ −0.198 [− 0.297; −0.099], *P* < 0.0001), and HbA_1c_ showing a modest association ($\:\widehat{{\upbeta\:}}$_SD_ −0.130 [− 0.262; 0.002], *P* = 0.053). In individuals with prediabetes and type 2 diabetes mellitus, HOMA-IR and HbA_1c_ showed relevant associations with HRR_60_ across models with varying levels of adjustment (age and sex only or additionally adjusted for complementary glucose or insulin status), while associations with HbA_1c_ were less consistent (see Supplementary Table 6).

In the subgroup of diabetic individuals, insulin therapy was associated with a lower HRR_60_ independently of sex, age, duration of type 2 diabetes, heart failure status, clinical profile, and medication ($\:\widehat{{\upbeta\:}}$_SD_ −0.343 [− 0.600; −0.086], *P* = 0.010, Supplementary Table 7). Metformin intake was only related to a higher HRR_60_ independently of sex and age, but not after further adjustments.

### C-peptide was a strong predictor of reduced vagal activity

Plasma insulin was less strongly related to HRR_60_ than HOMA-IR (Supplementary Table 8). However, C-peptide proved to be consistently associated with HRR_60_ in the analysis sample, across the heart failure stages, and type 2 diabetes spectrum (Table 3). The association with HRR_60_ remained independent of glucose metabolism or BMI ($\:\widehat{{\upbeta\:}}$_SD_ −0.17 [− 0.22; −0.11], *P* < 0.0001), and was present in both heart failure phenotypes independently of clinical profile (HFpEF (*n* = 394): $\:\widehat{{\upbeta\:}}$_SD_ −0.165 [− 0.258; −0.071], *P* < 0.001 and HFrEF (*n* = 268): $\:\widehat{{\upbeta\:}}$_SD_ −0.216 [− 0.335; −0.097], *P* < 0.001). Adjusting for HbA_1c_ did not affect the strength of the association (HFpEF: $\:\widehat{{\upbeta\:}}$_SD_ −0.150 [− 0.245; −0.055], *P* = 0.002 and HFrEF: $\:\widehat{{\upbeta\:}}$_SD_ −0.211 [− 0.332; −0.090], *P* < 0.001, Supplementary Table 9). Similar results were observed independently of maximal exertion and CPET protocol (Supplementary Fig. 2). Regarding proinsulin, it was associated with HRR_60_ in the analysis sample ($\:\widehat{{\upbeta\:}}$_SD_ −0.139 [− 0.205; −0.074], *P* < 0.001) and in the symptomatic HF subsample ($\:\widehat{{\upbeta\:}}$_SD_ −0.138 [− 0.222; −0.054], *P* = 0.0014), independently of clinical profile.

**Table 3:** Specific analysis on the relationship between C-peptide and HRR_60_ in different analysis samples

	Adjusted for sex and age		Additionally adjusted for HbA_1c_, cardiovascular risk factors, comorbidities, and medication	
	$\:\widehat{{\upbeta\:}}$-estimate per SD [95% CI]	*p*-value	$\:\widehat{{\upbeta\:}}$-estimate per SD [95% CI]	*p*-value
Total sample (*n* = 1,491)	−0.342 [− 0.389; −0.296]	< 0.0001	−0.171 [− 0.225; −0.117]	< 0.0001
*Heart failure Stages*				
Controls and at risk of heart failure (Stage 0/A) (*n* = 319)	−0.341 [− 0.456; −0.225]	< 0.0001	−0.274 [− 0.415; −0.133]	0.0002
Pre-heart failure (Stage B) (*n* = 510)	−0.282 [− 0.371; −0.192]	< 0.0001	−0.150 [− 0.259; −0.040]	0.0076
Symptomatic heart failure (Stage C/D) (*n* = 662)	−0.307 [− 0.373; −0.241]	< 0.0001	−0.173 [− 0.245; −0.101]	< 0.0001
*Type 2 diabetes mellitus spectrum*				
Euglycemia (*n* = 994)	−0.338 [− 0.410; −0.267]	< 0.0001	−0.182 [− 0.264; −0.101]	< 0.0001
Prediabetes (*n* = 204)	−0.326 [− 0.449; −0.203]	< 0.0001	−0.181 [− 0.320; −0.041]	0.012
Type 2 diabetes mellitus (*n* = 220)*	−0.230 [− 0.325; −0.135]	< 0.0001	−0.138 [− 0.239; −0.038]	0.0076

The $\:\widehat{{\upbeta\:}}$-estimates [per SD] presented are from multivariate regression analyses with C-peptide as independent variable and HRR_60_ as dependent variable. HRR_60_ is log-transformed. * Type 2 diabetic individuals with only dietary treatment were excluded from the analysis (*n* = 75). SD, standard deviation; CI, confidence interval

### Assessment of the relationship between insulin and glucose status and impaired vagal reactivity over time

In the analysis sample, higher levels of HOMA-IR, HbA_1c_, and C-peptide were consistently associated with lower HRR_60_ after two years of follow-up, with the strongest and most robust associations observed for HbA_1c_ and C-peptide, independently of sex, age, clinical profile, and medication (Table 4).

**Table 4:** Impact of HbA_1c_, HOMA-IR and C-peptide on HRR_60_ after two years in the whole sample and in the symptomatic heart failure sample

		Adjusted for age, sex, and baseline HRR_60_		Additionally adjusted for cardiovascular risk factors, comorbidities, and medication		Additionally adjusted for complementary glucose/insulin status^Φ^_1c_	
		$\:\widehat{{\upbeta\:}}$-estimate per SD [95% CI]	*p*-value	$\:\widehat{{\upbeta\:}}$-estimate per SD [95% CI]	p-value	$\:\widehat{{\upbeta\:}}$-estimate per SD [95% CI]	*p*-value
Whole sample	HbA_1c_	−0.177 [− 0.239; −0.115]	< 0.001	−0.105 [− 0.172; −0.038]	0.002	−0.094 [− 0.171; −0.017]	0.017
	HOMA-IR	−0.168 [− 0.233; −0.103]	< 0.001	−0.074, [− 0.149; 0.001]	0.053	−0.029 [− 0.112; 0.055]	0.50
	C-peptide	−0.200 [− 0.271; −0.129]	< 0.001	−0.096 [− 0.178; −0.013]	0.023	−0.076 [− 0.159; 0.007]	0.075
Symptomatic heart failure	HbA_1c_	−0.138 [− 0.257; −0.019]	0.024	−0.083 [− 0.209; 0.043]	0.20	−0.041 [− 0.192; 0.110]	0.59
	HOMA-IR	−0.147 [− 0.259; −0.034]	0.011	−0.088 [− 0.215; 0.040]	0.18	−0.067 [− 0.216; 0.083]	0.38
	C-peptide	−0.226 [− 0.359; −0.093]	0.001	−0.161 [− 0.311; −0.011]	0.037	−0.149 [− 0.302; 0.004]	0.057

The $\:\widehat{{\upbeta\:}}$-estimates [per SD] presented are from multivariate regression analyses in the whole sample (*n* = 757) and in symptomatic heart failure (*n* = 276). HbA_1c_, HOMA-IR, and C-peptide are the independent variables and HRR_60_ after two years of follow-up is the dependent variable. HRR_60_ is log-transformed. ^Φ^ Complementary glucose/insulin status indicates that HbA_1c_ was adjusted for HOMA-IR and vice versa. C-peptide was also adjusted for HbA_1c_.SD, standard deviation; CI, confidence interval

In individuals with symptomatic heart failure, C-peptide was most strongly associated with a reduction in HRR_60_ independently of all adjustment variables when considering effect estimates, while associations for HOMA-IR and HbA_1c_ attenuated with increasing model complexity (Table 4).

### Heart rate variability and metabolic dysregulation

In Supplementary Table 10, SDNN was most strongly related to hyperglycemia and both insulin markers. The LF/HF ratio showed the strongest association with C-peptide in the model adjusted for sex, age, and CPET protocol.

## Discussion

This project investigated the relationship between insulin and glucose status with heart rate recovery across stages of heart failure. Results in cross-sectional and sequential data, as well as in various clinical subgroups, consistently suggested that a hyperinsulinemic state, but not hyperglycemia, influences cardiac vagal autonomic function. While hyperglycemia was also associated with heart rate recovery, this relationship was no longer evident after adjusting for HOMA-IR, an established marker of insulin resistance. Conversely, HOMA-IR was consistently found to be negatively associated with HRR_60_ independently of HbA_1c_. C-peptide, as a marker of insulin metabolism, also proved to be a strong predictor of reduced cardiac parasympathetic activity. In individuals with heart failure, it was the only marker that suggested a reduced vagal activity after two years independently of clinical profile and medication.

These findings are even more remarkable given that fasting glucose and fasting insulin, on which the calculation of HOMA-IR is based, as well as C-peptide, are short-term markers unlike HbA_1c_. The measures can fluctuate over the course of the day, due to hormones or stress [24]. This could explain why the relationship between HOMA-IR and HRR_60_ was not as strong in pre-heart failure, in prediabetes nor in type 2 diabetes. However, another plausible explanation is that glucose metabolism is included in the calculation of the index, which could weaken the association between HOMA-IR and HRR_60_. Moreover, insulin measurement is affected by the intake of exogenous insulin therapy. Pure insulin is therefore not representative of actual endogenous insulin production in individuals treated with insulin [25, 26]. The strong inverse relationship between insulin and autonomic dysfunction was further supported by a similar association found between insulin therapy and HRR_60_. In individuals with type 2 diabetes mellitus and heart failure, insulin therapy, indicative of advanced disease [27], is known to be associated with increased cardiovascular complications [27], and a higher mortality risk [28, 29] .

Nonetheless, the finding that insulin metabolism affects HRR_60_, particularly in symptomatic heart failure, is not surprising as it has long been clear from pathophysiology and outcome data [30–32] that insulin impacts cardiovascular function. For instance, insulin resistance is characterized by inflammation markers as well as accumulation and dysfunction of the adipose tissue [33]. Such impairments are associated with heart failure and cardiovascular disease [34, 35]. Nitric oxide production is also mediated by insulin. In insulin resistant individuals, this production becomes inadequate, contributing to altered cardiac contractility [33, 36]. Regarding the relationship between insulin metabolism and cardiac autonomic dysfunction, an Italian study found that insulin resistant adult offspring of type 2 diabetic individuals presented autonomic dysfunction by an elevated ratio of low-frequency to high-frequency power (LF/HF ratio). This was measured during an intravenous glucose tolerance test to stimulate endogenous hyperinsulinemia [18]. Interestingly, the results were independent of glucose metabolism as all subjects presented with normal glucose tolerance. The results obtained in the current study emphasize the relevance of assessing insulin status as healthier subjects were also affected. Insulin is usually not part of routine evaluations in clinical practice. High insulin levels may thus go undetected, while HbA_1c_ is within the normal range [37]. 

Another important and surprising finding of this study was the strong relation between C-peptide and HRR_60_, indicating that it was more strongly associated with worsening cardiac vagal autonomic dysfunction per SD than HOMA-IR. As sensitivity analyses demonstrated, these results were not due to failure to reach peak heart rate or differences in the CPET protocol. These findings were also supported by a similar association between proinsulin and HRR_60_ as well as between insulin therapy and HRR_60_. While C-peptide avoids the aforementioned drawbacks of HOMA-IR, its longer half-life also makes it a more stable marker than insulin (approx. 30 min. compared to 3–5 min. for insulin) [25]. C-peptide is extracted by the kidneys and, unlike insulin, avoids hepatic extraction [25]. It correlated with glucose levels in fasted, healthy individuals with normal glucose tolerance while insulin did not [38]. This suggests that C-peptide better reflects the insulinemic response and may be a more robust insulin resistance marker than the classic marker HOMA-IR. Proinsulin, as another accurate and stable marker of insulin metabolism [25], further supported the findings. Emerging as another marker of interest, it was however measured in a smaller sample in the MyoVasc cohort and warrants further research.

Knowledge about the relationship between C-peptide and cardiovascular health outcomes has grown significantly in the past decade. C-peptide was found to be a strong predictor of cardiovascular mortality and complications in diseased and in nondiabetic individuals [26, 39, 40]. In the latter group, C-peptide was a better predictor than HOMA-IR, fasting glucose, fasting insulin as well as metabolic syndrome for both all-cause mortality and cardiovascular death, highlighting that C-peptide is predictive of outcomes even in healthier individuals with lower cardiovascular risk [26]. Clinically, higher insulin resistance has been observed in heart failure, with HFrEF patients also presenting worse glucose clearance than HFpEF subjects [41]. An important mechanism is related to the disturbance of the insulin signaling pathways, which can lead to proliferation and migration of vascular smooth muscle cells and indirectly contribute to heart failure [42, 43]. For instance, the PI3K-Akt pathway is known to lead to cardiac hypertrophy and fibrosis through altered angiogenesis and cell proliferation at the cardiovascular level. Disrupted insulin pathways also cause elevated free fatty acids levels, which further impede cardiac function and enhance cardiac inflammation [44, 45]. The impact of C-peptide on autonomic dysfunction, however, has not yet been well characterized in literature. A 1997 study observed a significant impact of C-peptide on heart rate variability derived from arterial pressure in the middle finger [46]. Fasting plasma C-peptide was negatively associated with the MF/HF ratio (medium frequency band: 0.07 to 0.15 Hz, similar to the low frequency band) in both non-diabetic and non-insulin dependent diabetic individuals (*N* = 165), independently of fasting glucose [46]. Moreover, C-peptide explained 24.2% of the MF/HF ratio variability in diabetic patients as opposed to 10.5% for proinsulin [46]. This suggests an important role for C-peptide in autonomic dysfunction in individuals with and without diabetes. This was also found in the current project under better controlled conditions and with a larger sample size. C-peptide was more strongly related to the LF/HF ratio. However, this was not the case for SDNN or RMSSD, and although research on the topic has already found heart rate variability to be affected by insulin resistance [47], and diabetes mellitus or prediabetes [48], further research is necessary. Furthermore, sequential analyses revealed that C-peptide may independently be related to a deterioration in autonomic function over two years, regardless of clinical profile and HbA_1c_, in subjects with heart failure, highlighting the importance of hyperinsulinemia in the progression of this complex syndrome. Additional analyses with other heart rate variability measures or baroreflex sensitivity in the MyoVasc cohort could provide a more complete picture of the relationship between cardiac autonomic function and metabolic regulation.

HbA_1c_ was also associated with a change in parasympathetic activity over a two-year period in the whole sample, indicating that the long-term marker HbA_1c_ performed better in sequential than in cross-sectional analyses. CPET tests over time may lead to slightly varying results, as found in a study conducted in the UK Biobank with a correlation of 0.70 between two CPET tests performed at least three years apart; the reproducibility of abnormal HRR_60_ (probability to remain in the lowest quintile during the second CPET) was 65% [49]. 

One of the strengths of the project is the highly standardized cohort, which is one of the few in which such a large number of patients underwent prospective CPET. The fine-grained investigation with extensive data enabled in-depth analyses in specific subgroups. Due to physical and technical limitations, approx. 1,000 individuals were however unable to perform CPET. Moreover, fewer participants performed CPET during the follow-up examination, potentially inducing a selection bias, as it is more likely that sicker individuals did not undergo the examination. Because of the assessment time of the study with long-term follow-up, there were only very low numbers for newer antidiabetic drugs like SGLT2 inhibitors. This was not the primary research question of this study, but these drugs have been shown to reduce the risk of heart failure in people with type 2 diabetes mellitus [50, 51]. They might affect autonomous dysfunction by targeting insulin resistance, a topic which could be addressed in future research. Finally, all analyses, in particular subgroup and sequential analyses, were exploratory in nature and should be interpreted as hypothesis-generating.

## Conclusions

In this large heart failure cohort, reduced cardiac vagal activity, assessed by heart rate recovery after physical exertion, was consistently and strongly associated with insulin metabolism. While hyperglycemia was also linked to impaired parasympathetic reactivation, this association was no longer present after adjusting for insulin status, indicating that disturbed glucose metabolism largely reflects underlying insulin resistance or hyperinsulinemia. C-peptide, as another stable marker of insulin metabolism, emerged as an especially strong and independent predictor of cardiac autonomic dysfunction. These findings underscore the central role of insulin metabolism in driving cardiac autonomic dysfunction and highlight the potential value of targeting insulin resistance to preserve vagal function and improve clinical outcomes in heart failure.

## Supplementary Information

Below is the link to the electronic supplementary material.

Supplementary Material 1

## Data Availability

The clinical data in this study, including CPET assessment and laboratory markers, are under controlled access, as per the signed study participants’ consent. Request for access to data can be addressed to the principal investigators of the study.

## Abbreviations

CI: Confidence interval
CPET: Cardiopulmonary exercise testing
HFpEF: Heart failure with preserved ejection fraction
HFrEF: Heart failure with reduced ejection fraction
HRR_60_: Heart rate recovery after 60 seconds
IQR: Interquartile range
LVEF: Left ventricular ejection fraction
NYHA: New York Heart Association
RER: Respiratory exchange ratio
SD: Standard deviation
