# Supplementary material for: Cardiac vagal activity is associated with insulin metabolism in heart failure: Results from the Myovasc study
Source: Cardiovasc Diabetol. 2026 Jan 8;25:26. doi: 10.1186/s12933-025-03040-9 (PMC12849475; doi:10.1186/s12933-025-03040-9)
Supplement: Supplementary file 1 — Supplementary Material 1 [file 12933_2025_3040_MOESM1_ESM.docx]

**SUPPLEMENTAL APPENDIX**

**Cardiac vagal activity is associated with insulin metabolism in heart failure – Results from the MyoVasc study**

Noémie Bélanger (0009-0001-9267-8788)^1,2^, Silav Zeid^1,2^, David Velmeden (0009-0006-3714-312X)^1,2^, Andreas Schulz (0000-0003-1394-680X)^1^, Thomas Koeck (0000-0002-2644-8917)^1,2^, Felix Rausch (0000-0002-7328-1056)^1,2^, Benedikt Fooß^1,2^, Fawad Kazemi-Asrar (0009-0006-8245-8243)^1,2^, Karl J. Lackner (0000-0002-1985-7931)^3,2^, Tommaso Gori (0000-0003-2917-6707)^2,4^, Thomas Münzel (0000-0001-5503-4150)^2,4^, Jürgen H. Prochaska (0000-0001-5078-9992)^1,6^, Perikles Simon^7^ (000-0002-7996-4034), Philipp S. Wild (0000-0003-4413-9752)^1,2,5,8^

^1^Preventive Cardiology and Preventive Medicine, Department of Cardiology, University Medical Center of the Johannes Gutenberg University Mainz, Germany;

^2^German Center for Cardiovascular Research (DZHK), partner site Rhine-Main, Mainz, Germany;

^3^Institute for Clinical Chemistry and Laboratory Medicine, University Medical Center of the Johannes Gutenberg University Mainz, Germany;

^4^Department of Cardiology – Cardiology I, University Medical Center of the Johannes Gutenberg University Mainz, Germany;

^5^Clinical Epidemiology and Systems Medicine, Center for Thrombosis and Hemostasis (CTH), University Medical Center of the Johannes Gutenberg University Mainz, Germany;

^6^Boehringer Ingelheim International GmbH, Ingelheim, Germany;

^7^Sports Medicine and Disease Prevention, Department of Sports Medicine, Prevention and Rehabilitation, [Johannes Gutenberg-University Mainz](https://scholar.google.com/citations?view_op=view_org&hl=de&org=4253128451676025523), Germany;

^8^Systems Medicine, Institute of Molecular Biology (IMB), Mainz, Germany.

**Address for correspondence**

Philipp S. Wild, MD, MSc

Professor of Clinical Epidemiology

Preventive Cardiology and Preventive Medicine, Department of Cardiology and

Clinical Epidemiology and Systems Medicine, Center for Thrombosis and Hemostasis

University Medical Center of the Johannes Gutenberg-University Mainz

Langenbeckstr. 1, 55131 Mainz, Germany

Phone: +49 6131 17 7163

Fax: +49 6131 17 8460

Email: philipp.wild@unimedizin-mainz.de

**Table of Contents**

[**Supplementary methods** 3](#_Toc199451541)

[**Supplementary figures and tables** 4](#_Toc199451542)

[Details on the medication score 4](#_Toc199451543)

[Supplementary Figure 1 caption 5](#_Toc199451544)

[Supplementary Figure 2 caption 5](#_Toc199451545)

[Supplementary Figure 1. Flow chart of excluded and included individuals in the study 5](#_Toc199451546)

[Supplementary Figure 2. Sensitivity analysis in a subsample that achieved a respiratory exchange ratio>1.0 across the heart failure stages and the type 2 diabetes mellitus spectrum……………………………………………………………………………………………..6](#_Toc199451547)

[Supplementary Table 1. Additional baseline characteristics of the sample 7](#_Toc199451548)

[Supplementary Table 2. Baseline characteristics of the whole sample according to the heart failure stages 9](#_Toc199451549)

[Supplementary Table 3. Comparison of glucose and insulin status between ≥8 and ≥5 hours of fasting according to the heart failure stages 12](#_Toc199451550)

[Supplementary Table 4. Relationship between glucose and insulin status and HRR_60_ across the heart failure stages (other models not previously shown) 13](#_Toc199451551)

[Supplementary Table 5. Heart failure phenotype-specific analysis between insulin and glucose status and HRR_60_ 15](#_Toc199451552)

[Supplementary Table 6. Influence of type 2 diabetic state on the relationship between glucose and insulin status and HRR_60_ (other models not previously shown) 16](#_Toc199451553)

[Supplementary Table 7. Effect of metformin and insulin therapy on HRR_60_ in individuals with type 2 diabetes 17](#_Toc199451554)

[Supplementary Table 8. Specific analysis on the relationship between insulin and HRR_60_ in different analysis samples 18](#_Toc199451555)

[Supplementary Table 9. Specific analysis on the relationship between C-peptide and HRR_60_ in different analysis samples 20](#_Toc199451556)

[Supplementary Table 10. Additional analysis on with heart rate variability parameters 21](#_Toc199451556)

# **Supplementary methods**

*Heart failure Stage C/D definition*

The heart failure sample (stage C/D) includes patients with current or prior symptoms and/or signs of HF caused by structural and/or functional cardiac abnormality (stage C) and with severe symptoms and/or signs of HF at rest, recurrent hospitalizations despite optimized medical therapy, or need for advanced interventions (e.g., LVAD, transplant, palliative care) for stage D as stated in the Universal Definition and Classification of Heart Failure published in 2021.

*Exercise testing protocols*

Patients in New York Heart Association (NYHA) functional classes I or II would take a modified Jones protocol (WHO-50), which started at 50W with progressive 25W increments every 3 minutes. Patients in NYHA functional classes III to IV underwent the WHO-25, with a starting workload of 20W and 10W increments, also every 3 minutes. An unloaded 2-minute warm-up period (for the WHO-25 protocol) or with a 25W load (for the WHO-50 protocol) preceded the test.

*Termination criteria*

Other absolute criteria for CPET termination included: 1) a blood pressure (BP) decrease ≥10 mmHg compared to the resting BP despite incremental workload and additional signs of ischemia, 2) angina pectoris or sudden-onset dyspnea, 3) neurologic symptoms such as ataxia or vertigo, 4) signs of poor peripheral perfusion such as cyanosis or sudden paleness, 5) persistent ventricular tachycardia, and 6) ST-segment elevation >1 mm without Q−waves. The physician in charge could also terminate the exercise if patients would present relative termination criteria: a) a BP decrease ≥10 mmHg compared to the resting BP despite incremental workload without additional signs of ischemia, b) ST-segment alterations >2 mm (horizontal or descending), and QRS broadening, c) physical exhaustion, d) branch block not classified as ventricular tachycardia, and e) systolic BP >250 mmHg or diastolic BP >115 mmHg.

**Supplementary results**

## Details on the medication score

Medication was derived from a medication score based on medication with an impact on HRR_60_ (Bonferroni threshold = 0.00065, adjusted for sex and age) and comprised β-blocking agents [ATC code: C07A], antithrombotic agents [B01A], drugs for peptic ulcer and GERD [A02B], antigout preparations [M04A], high-ceiling diuretics [C03C], insulins and analogues [A10A], antiarrhythmics [C01B], potassium-sparing agents [C03D], adrenergics, inhalants [R03A], blood glucose lowering drugs, excluding insulins [A10B], selective calcium channel blockers with mainly vascular effects [C08C], other cardiac preparations [C01E], angiotensin-converting enzyme inhibitors [C09B], opioids [N02A], other drugs for obstructive airway diseases, inhalants [R03B], antiepileptics [N03A], lipid modifying agents [C10A], antiadrenergic agents, peripherally acting [C02C], antidepressants [N06A], and corticosteroids for systemic use, plain [H02A].

**Supplementary figures and tables**

## Supplementary Figure 1. Flow chart of excluded and included individuals in the study


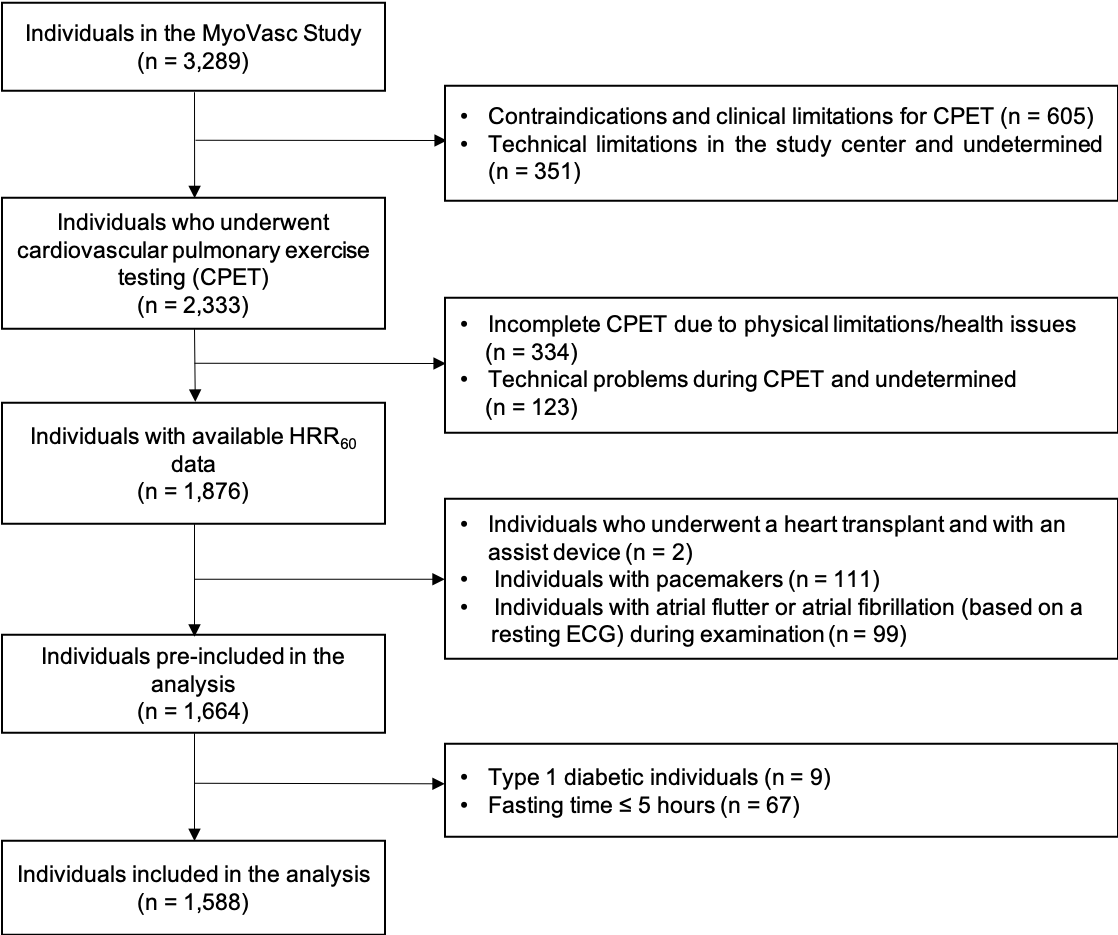


A total of 1,588 individuals fasting for 5 hours or more from the MyoVasc study were included in the analysis.

## Supplementary Figure 2. Sensitivity analysis in a subsample that achieved a respiratory exchange ratio>1.0 across the heart failure stages and the type 2 diabetes mellitus spectrum


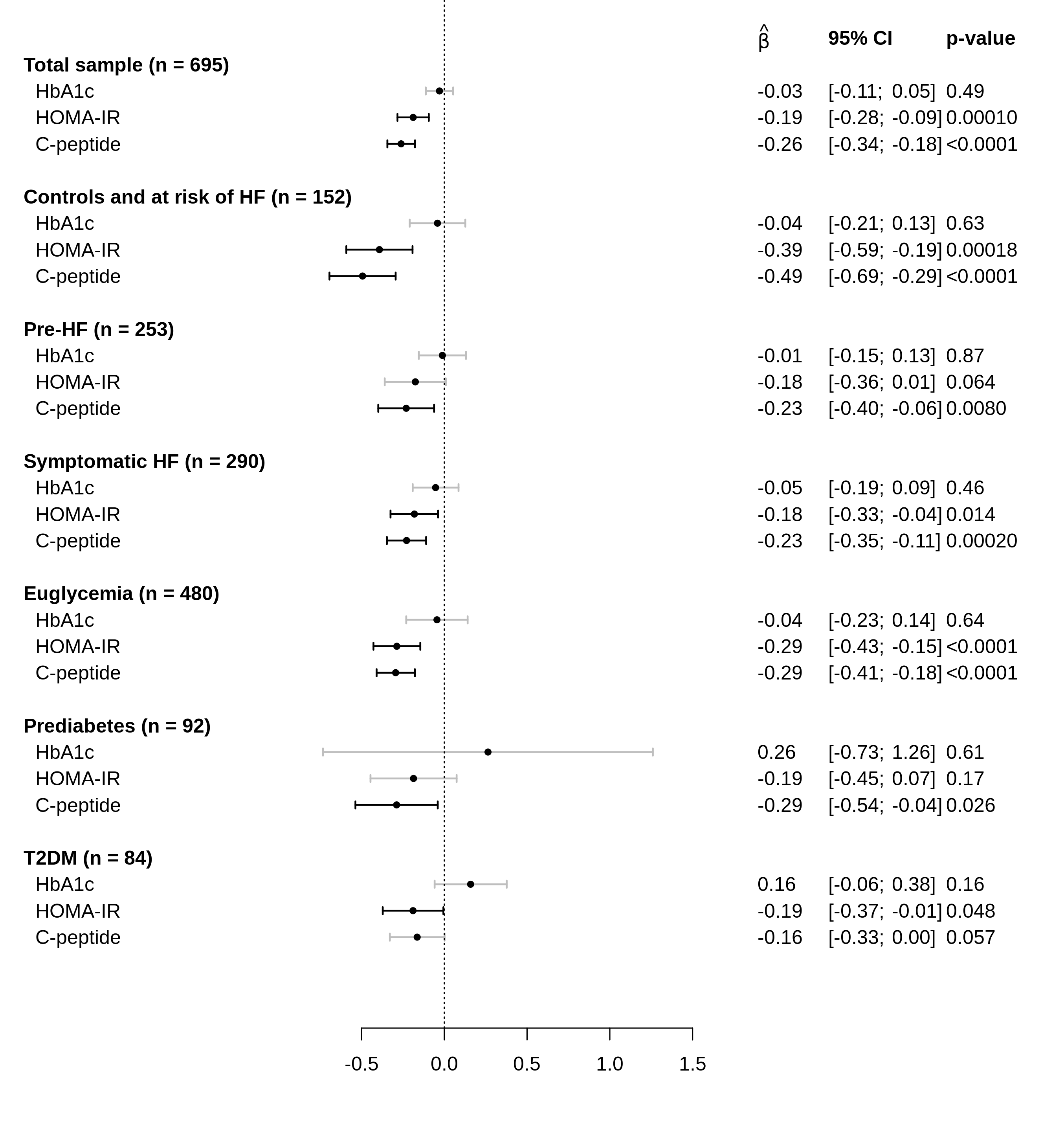


Heart failure stages

Type 2 diabetes spectrum

*

The $\hat{\beta}$-estimates [per SD] presented are from multivariate regression analyses with HbA_1c_, HOMA-IR, and C-peptide as independent variables and HRR_60_ as dependent variable. HRR_60_ is log-transformed. Models are performed in a subsample that achieved maximal exertion and are adjusted for cardiopulmonary exercise testing protocol, cardiovascular risk factors, comorbidities, and medication as previously described, as well as complementary glucose/insulin status indicates that HbA_1c_ was adjusted for HOMA-IR and vice versa. C-peptide was also adjusted for HbA_1c_. * Type 2 diabetic individuals with only dietary treatment were excluded from the analysis (n = 39). HF, heart failure; SD, standard deviation; CI, confidence interval.

## Supplementary Table 1. Additional baseline characteristics of the sample

| **Characteristics** |  |
| --- | --- |
| *n* | 1,588 |
| NT-pro-BNP in pg/ml, median (IQR) | 116.5 (58.0−267.6) |
| Left ventricular ejection fraction in %, median (IQR) | 57.7 (51.3−62.5) |
| Other antidiabetic medication, % (n) |  |
| Sulfonylureas (ATC code: A10BB) | 1.1 (17) |
| DPP-4 inhibitors (A10BH) | 1.6 (26) |
| GLP-1 analogues (A10BJ) | 0.5 (8) |
| SGLT2 inhibitors (A10BK) | 0.6 (9) |
| Other medication, % (n) |  |
| Antithrombotic agents (B01A) | 57.4 (911) |
| Cardiac therapy (C01) | 13.6 (216) |
| Antihypertensives (C02) | 2.2 (35) |
| Diuretic agents (C03) | 23.7 (377) |
| β-receptor blocking agents (C07) | 50.6 (803) |
| Calcium channel blocker (C08) | 16.9 (268) |
| Angiotensin-converting enzyme inhibitors (C09A and C09B) | 33.6 (533) |
| Lipid modifying agents (C10) | 45.8 (727) |
| Antigout preparations (M04) | 8.4 (133) |
| Antidepressants (N06A) | 7.4 (118) |
| Drugs for obstructive airway diseases (R03) | 10.1 (161) |

NT-pro-BNP, N-terminal pro-B-type natriuretic peptide; IQR, interquartile range; DPP-4, dipeptidyl peptidase-4; GLP-1, glucagon-like peptide 1; SGLT2, sodium-glucose cotransporter-2.

## Supplementary Table 2. Baseline characteristics of the whole sample according to the heart failure stages

| **Characteristics** | **Controls and at risk of heart failure (Stage 0/A)** | **Pre-heart failure (Stage B)** | **Symptomatic heart failure (Stage C/D)** | |
| --- | --- | --- | --- | --- |
| *n* | 363 | 531 | 694 | |
| Age in years | 55.0 (47.0−63.0) | 65.0 (56.0−72.0) | 68.0 (59.9−74.0) | |
| Sex, % women (n) | 41.6 (151) | 30.3 (161−531) | 30.5 (212) | |
| BMI in kg/m² | 26.4 (23.9−29.4) | 27.1 (24.6−29.7) | 28.3 (25.4−31.9) | |
| Heart failure-related characteristics |  |  |  | |
| NT-pro-BNP in pg/ml | 51.0 (32.2−77.8) | 99.0 (56.0−179.0) | 253.0 (142.4−559.9) | |
| Left ventricular ejection fraction in % | 61.4 (58.3−65.4) | 58.3 (54.3−63.5) | 53.3 (44.1−59.2) | |
| HRR_60_ at baseline in bpm | 25.0 (20.0−31.0) | 21.0 (16.0−28.0) | 17.00 (10.7−24.0) | |
| NYHA functional classification, % (n) |  |  |  | |
| NYHA I | 88.7 (322) | 95.8 (507) | 51.6 (358) | |
| NYHA II | 8.0 (29) | 2.5 (13) | 34.4 (239) | |
| NYHA III and IV | 3.3 (12) | 1.7 (9) | 14.0 (97) | |
| Glucose and insulin status |  |  |  | |
| Type 2 diabetes mellitus (%) | 7.2 (26) | 17.1 (91) | 26.7 (185) | |
| HbA_1c_ in % | 5.50 (5.20−5.76) | 5.60 (5.40−6.00) | 5.80 (5.50−6.20) | |
| HOMA-IR | 1.23 (0.82−1.81) | 1.57 (1.02−2.41) | 1.91 (1.30−3.17) | |
| C-peptide in ng/mL | 1.49 (1.17−1.95) | 1.69 (1.29−2.32) | 2.12 (1.54−2.95) | |
| Proinsulin in pmol/l | 3.73 (2.10−5.25) | 3.72 (2.41−5.70) | 4.23 (2.72−7.33) | |
| Antidiabetic medication, % (n) | 5.8 (21) | 11.1 (59) | 21.2 (147) | |
| Insulins and analogues | 1.1 (4) | 3.8 (20) | 7.2 (50) | |
| Biguanides | 3.9 (14) | 5.6 (30) | 12.0 (83) | |
| Cardiovascular risk factors, % (n) |  |  |  | |
| Arterial hypertension | 49.9 (181) | 67.4 (358) | 78.7 (546) | |
| Dyslipidemia | 47.1 (171) | 67.6 (359) | 81.4 (565) | |
| Family history of myocardial  infarction/stroke | 19.8 (72) | 21.4 (113) | 24.8 (172) | |
| Obesity | 20.9 (76) | 23.7 (126) | 34.4 (239) | |
| Smoking in the last 7 years | 17.4 (63) | 22.2 (118) | 26.5 (184) | |
| Comorbidities, % (n) |  |  |  | |
| Atrial fibrillation based on anamnesis | 5.5 (20) | 14.1 (75) | 22.0 (153) | |
| Chronic kidney disease, eGFR<  60ml/min/1.73 m^2^ | 1.7 (6) | 11.7 (62) | 21.2 (146) | |
| Chronic obstructive pulmonary disease | 14.9 (54) | 15.8 (84) | 26.1 (181) | |
| Coronary artery disease | 9.1 (33) | 33.5 (178) | 54.2 (376) | |
| History of cancer | 13.2 (48) | 12.2 (65) | 17.9 (124) | |
| History of myocardial infarction | 0 | 21.7 (115) | 39.0 (271) | |
| History of stroke | 3.9 (14) | 7.5 (40) | 8.8 (61) |  |
| Peripheral artery disease | 0.8 (3) | 3.4 (18) | 8.2 (57) |  |
| Venous thromboembolism | 4.4 (16) | 6.6 (35) | 9.2 (64) |  |

All continuous variables are presented as the median with the interquartile range. BMI, body mass index; HRR_60_, heart rate recovery at 60 seconds post-exercise; bpm, beats per minute; NYHA, New York Heart Association; eGFR, estimated glomerular filtration rate.

## Supplementary Table 3. Comparison of glucose and insulin status between ≥8 and ≥5 hours of fasting according to the heart failure stages

| **Marker, median (IQR)** | **Controls and at risk of heart failure (Stage 0/A)** | | **Pre-heart failure (Stage B)** | | **Symptomatic heart failure (Stage C/D)** | |
| --- | --- | --- | --- | --- | --- | --- |
|  | ≥8 hours  (n = 273) | ≥5 hours  (n = 363) | ≥8 hours  (n = 414) | ≥5 hours  (n = 531) | ≥8 hours  (n = 544) | ≥5 hours  (n = 694) |
| Plasma HbA_1c_ (%) | 5.50 (5.30−5.80) | 5.50 (5.20−5.76) | 5.70 (5.40−6.10) | 5.60 (5.40−6.00) | 5.90 (5.60−6.20) | 5.80 (5.50−6.20) |
| Plasma glucose (mg/dL) | 89.0 (84.0−98.0) | 89.0 (84.0−97.0) | 96.0 (88.0−105.0) | 95.0 (88.0−102.0) | 100.0 (92.0−110.3) | 98.0 (91.0−109.0) |
| Serum insulin (pmol/L) | 5.60 (4.07−8.53) | 5.20 (3.82−7.78) | 6.70 (4.80−10.33) | 6.50 (4.60−9.90) | 8.20 (5.80−12.30) | 7.90 (5.50−12.00) |
| HOMA-IR | 1.30 (0.86−1.98) | 1.23 (0.82−1.81) | 1.64 (1.12−2.56) | 1.57 (1.02−2.41) | 2.03 (1.39−3.28) | 1.91 (1.30−3.17) |
| Serum proinsulin (pmol/L) | 3.65 (2.10−5.02) | 3.73 (2.10−5.17) | 3.74 (2.45−5.72) | 3.71 (2.41−5.74) | 4.33 (2.79−7.11) | 4.18 (2.71−7.07) |
| Serum C-peptide (ng/mL) | 1.56 (1.21−2.01) | 1.49 (1.17−1.95) | 1.77 (1.34−2.42) | 1.69 (1.29−2.32) | 2.20 (1.62−3.02) | 2.12 (1.54−2.95) |

IQR, interquartile range.

## Supplementary Table 4. Relationship between glucose and insulin status and HRR_60_ across the heart failure stages (other models not previously shown)

|  | **Controls and at risk of heart failure (Stage 0/A)** **(n = 360)** | | **Pre-heart failure (Stage B)**  **(n = 529)** | | **Symptomatic heart failure (Stage C/D)**  **(n = 686)** | | |
| --- | --- | --- | --- | --- | --- | --- | --- |
|  | Adjusted for sex and age | Additionally adjusted for complementary glucose/insulin status^Φ^ | Adjusted for sex and age | Additionally adjusted for complementary glucose/insulin status | Adjusted for sex and age | Additionally adjusted for complementary glucose/insulin status | |
|  | $\hat{\beta}$-estimate per SD [95% CI] | $\hat{\beta}$-estimate per SD [95% CI] | $\hat{\beta}$-estimate per SD [95% CI] | $\hat{\beta}$-estimate per SD [95% CI] | $\hat{\beta}$-estimate per SD [95% CI] | | $\hat{\beta}$-estimate per SD [95% CI] |
| HbA_1c_ | −0.133 [−0.238; −0.028], p=0.014 | −0.062 [−0.174; 0.050], p=0.28 | −0.160 [−0.237; −0.083], p<0.001 | −0.066 [−0.158; 0.027], p=0.17 | −0.215 [−0.288; −0.142], p<0.001 | −0.082 [−0.170; 0.005], p=0.067 | |
| HOMA-IR | −0.329 [−0.454; −0.204], p<0.001 | −0.306 [−0.438; −0.175], p<0.001 | −0.218 [−0.302; −0.134], p<0.001 | −0.179 [−0.280; −0.078], p<0.001 | −0.250 [−0.317; −0.182], p<0.001 | −0.209 [−0.290; −0.127], p<0.001 | |

The $\hat{\beta}$-estimates [per SD] presented are from multivariate regression analyses with HbA_1c_ and HOMA-IR as independent variables and HRR_60_ as dependent variable. HRR_60_ is log-transformed. ^Φ^ Complementary glucose/insulin status indicates that HbA_1c_ was adjusted for HOMA-IR and vice versa. SD, standard deviation; CI, confidence interval.

## Supplementary Table 5. Heart failure phenotype-specific analysis between insulin and glucose status and HRR_60_

|  | **HFpEF (n = 411)** | | | | **HFrEF (n = 275)** | | |
| --- | --- | --- | --- | --- | --- | --- | --- |
|  | Adjusted for sex and age | Additionally adjusted for cardiovascular risk factors, comorbidities, and medication | Additionally adjusted for complementary glucose/insulin status^Φ^ | Adjusted for sex and age | | Additionally adjusted for cardiovascular risk factors, comorbidities, and medication | Additionally adjusted for complementary glucose/insulin status^Φ^ |
|  | $\hat{\beta}$-estimate per SD [95% CI] | $\hat{\beta}$-estimate per SD [95% CI] | $\hat{\beta}$-estimate per SD [95% CI] | $\hat{\beta}$-estimate per SD [95% CI] | | $\hat{\beta}$-estimate per SD [95% CI] | $\hat{\beta}$-estimate per SD [95% CI] |
| HbA_1c_ | −0.228 [−0.319;  −0.137], p<0.001 | −0.086 [−0.179; 0.008], p=0.073 | −0.038 [−0.141; 0.065], p=0.47 | −0.190 [−0.309;  −0.071], p=0.002 | | −0.082 [−0.199; 0.036], p=0.17 | −0.009 [−0.149; 0.132], p=0.90 |
| HOMA-IR | −0.275 [−0.362;  −0.188], p<0.001 | −0.144 [−0.237;  −0.052], p=0.002 | −0.122 [−0.225;  −0.019], p=0.021 | −0.209 [−0.317;  −0.101], p<0.001 | | −0.104 [−0.215; 0.007], p=0.068 | −0.100 [−0.230; 0.031], p=0.13 |

The $\hat{\beta}$-estimates [per SD] presented are from multivariate regression analyses with HbA_1c_ and HOMA-IR as independent variables and HRR_60_ as dependent variable. HRR_60_ is log-transformed. The heart failure phenotypes were defined according to the left ventricular ejection fraction [LVEF] (HFpEF: LVEF>50% and HFrEF: ≤50%). ^Φ^ Complementary glucose/insulin status indicates that HbA_1c_ was adjusted for HOMA-IR and vice versa. SD, standard deviation; CI, confidence interval.

## Supplementary Table 6. Influence of type 2 diabetic state on the relationship between glucose and insulin status and HRR_60_ (other models not previously shown)

|  | **Euglycemia**  **(n = 1,060)** | | **Prediabetes**  **(n = 214)** | | **Type 2 diabetes on antidiabetic medication** **(n = 226)** | |
| --- | --- | --- | --- | --- | --- | --- |
|  | Adjusted for sex and age | Additionally adjusted for complementary glucose/insulin status^Φ^ | Adjusted for sex and age | Additionally adjusted for  complementary glucose/insulin status^Φ^ | Adjusted for sex and age | Additionally adjusted for complementary glucose/insulin status^Φ^ |
|  | $\hat{\beta}$-estimate per SD [95% CI] | $\hat{\beta}$-estimate per SD [95% CI] | $\hat{\beta}$-estimate per SD [95% CI] | $\hat{\beta}$-estimate per SD [95% CI] | $\hat{\beta}$-estimate per SD [95% CI] | $\hat{\beta}$-estimate per SD [95% CI] |
| HbA_1c_ | −0.255 [−0.387;  −0.123], p<0.001 | −0.186 [−0.323;  −0.050], p=0.007 | 0.164 [−0.527; 0.855], p=0.64 | 0.363 [−0.347; 1.07], p=0.32 | −0.134 [−0.244;  −0.024], p=0.017 | −0.014 [−0.131; 0.103], p=0.81 |
| HOMA-IR | −0.332 [−0.426;  −0.239], p<0.001 | −0.312 [−0.408;  −0.216], p<0.001 | −0.255 [−0.424;  −0.087], p=0.003 | −0.263 [−0.433;  −0.094], p=0.003 | −0.185 [−0.264;  −0.107], p<0.001 | −0.181 [−0.267;  −0.096], p<0.001 |

The $\hat{\beta}$-estimates [per SD] presented are from multivariate regression analyses with HbA_1c_ and HOMA-IR as independent variables and HRR_60_ as dependent variable. HRR_60_ is log-transformed. ^Φ^ Complementary glucose/insulin status indicates that HbA_1c_ was adjusted for HOMA-IR and vice versa. SD, standard deviation; CI, confidence interval.

## Supplementary Table 7. Effect of metformin and insulin therapy on HRR_60_ in individuals with type 2 diabetes

| **Antidiabetic medication^Φ^** | **Adjusted for sex, age,**  **and duration of type 2 diabetes** | | **Additionally adjusted for**  **NT-proBNP and NYHA class** | | **Additionally adjusted for medication, cardiovascular risk factors and comorbidities** | |
| --- | --- | --- | --- | --- | --- | --- |
|  | $\hat{\beta}$-estimate [95% CI] | p-value | $\hat{\beta}$-estimate [95% CI] | p-value | $\hat{\beta}$-estimate [95% CI] | p-value |
| Metformin | 0.271 [0.019; 0.524] | 0.036 | 0.212 [−0.027; 0.450] | 0.083 | 0.046 [−0.191; 0.284] | 0.70 |
| Insulin | −0.493 [−0.774; −0.211] | <0.001 | −0.390 [−0.655; −0.124] | 0.004 | −0.343 [−0.600; −0.086] | 0.010 |

The $\hat{\beta}$-estimates presented are from multivariate regression analyses in individuals with type 2 diabetes (n = 239) with metformin and insulin as independent variables and HRR_60_ as dependent variable. HRR_60_ is log-transformed. ^Φ^ All models are adjusted for metformin/insulin to account for individuals taking both therapies. Cardiovascular risk factors, comorbidities, and medication are the same as previously described, but antidiabetic medication is excluded from the score. NT-proBNP, N-terminal pro-B-type natriuretic peptide; NYHA, New York Heart Association; SD, standard deviation; CI, confidence interval.

## Supplementary Table 8. Specific analysis on the relationship between insulin and HRR_60_ in different analysis samples

|  | **Adjusted for sex and age** | **Additionally adjusted for cardiovascular risk factors, comorbidities, and medication** | **Additionally adjusted for HbA_1c_** |
| --- | --- | --- | --- |
|  | $\hat{\beta}$-estimate per SD [95% CI] | $\hat{\beta}$-estimate per SD [95% CI] | $\hat{\beta}$-estimate per SD [95% CI] |
| Total sample (n = 1,491) | −0.284 [−0.332; −0.237], p<0.001 | −0.125 [−0.177; −0.073], p<0.001 | −0.111 [−0.166; −0.056], p<0.001 |
| *Heat failure stages* |  |  |  |
| Controls and at risk of heart  failure (Stage 0/A) (n = 319) | −0.342 [−0.455; −0.229], p<0.001 | −0.278 [−0.415; −0.142], p<0.001 | −0.273 [−0.410; −0.135], p<0.001 |
| Pre-heart failure (Stage B)  (n = 510) | −0.221 [−0.303; −0.140], p<0.001 | −0.100 [−0.196; −0.003], p=0.043 | −0.091 [−0.191; 0.009], p=0.076 |
| Symptomatic heart failure  (Stage C/D)  (n = 662) | −0.253 [−0.323; −0.183], p<0.001 | −0.135 [−0.206; −0.063], p<0.001 | −0.120 [−0.196; −0.043], p=0.002 |
| *Type 2 diabetes spectrum* |  |  |  |
| Euglycemia (n = 994) | −0.293 [−0.370; −0.215], p<0.001 | −0.193 [−0.275; −0.111], p<0.001 | −0.181 [−0.265; −0.098], p<0.001 |
| Prediabetes (n = 204) | −0.234 [−0.380; −0.088], p=0.002 | −0.071 [−0.221; 0.078], p=0.35 | −0.078 [−0.228; 0.071], p=0.31 |
| Type 2 diabetes (n = 220)* | −0.200 [−0.285; −0.115], p<0.001 | −0.099 [−0.188; −0.009], p=0.031 | −0.092 [−0.183; −0.000], p=0.050 |

The $\hat{\beta}$-estimates [per SD] presented are from multivariate regression analyses with insulin as independent variable and HRR_60_ as dependent variable. HRR_60_ is log-transformed. The models are adjusted for sex, age, medication, cardiovascular risk factors, and comorbidities.

* Type 2 diabetic individuals with only dietary treatment were excluded from the analysis (n = 75). Cardiovascular risk factors, comorbidities, and medication are the same as previously described.

SD, standard deviation; CI, confidence interval.

## Supplementary Table 9. Specific analysis on the relationship between C-peptide and HRR_60_ in different analysis samples

|  | $\hat{\boldsymbol{\beta}}$**-estimate per SD [95% CI]** | **p-value** |
| --- | --- | --- |
| Total sample (n = 1,491) | −0.181 [−0.234; −0.127] | <0.001 |
| *Heart failure Stages* |  |  |
| Controls and at risk of heart failure (Stage 0/A) (n = 319) | −0.279 [−0.420; −0.139] | <0.001 |
| Pre-heart failure (Stage B) (n = 510) | −0.156 [−0.261; −0.050] | 0.004 |
| Symptomatic heart failure (Stage C/D) (n = 662) | −0.182 [−0.253; −0.111] | <0.001 |
| *Type 2 diabetes spectrum* |  |  |
| Euglycemia (n = 994) | −0.194 [−0.275; −0.114] | <0.001 |
| Prediabetes (n = 204) | −0.172 [−0.312; −0.033] | 0.016 |
| Type 2 diabetes (n = 220)* | −0.135 [−0.235; −0.034] | 0.009 |

The $\hat{\beta}$-estimates [per SD] presented are from multivariate regression analyses with C-peptide as independent variable and HRR_60_ as dependent variable. HRR_60_ is log-transformed. The models are adjusted for sex, age, cardiovascular risk factors, comorbidities, and medication. Cardiovascular risk factors, comorbidities, and medication are the same as previously described. *Type 2 diabetic individuals with only dietary treatment were excluded from the analysis (n = 75). SD, standard deviation; CI, confidence interval.

## Supplementary Table 10. Additional analysis on with heart rate variability parameters

|  |  | **Adjusted for sex, age, and CPET protocol** | | **Additionally adjusted for cardiovascular risk factors, comorbidities, and medication** | |
| --- | --- | --- | --- | --- | --- |
|  |  | $\hat{\beta}$-estimate per SD [95% CI] | p-value | $\hat{\beta}$-estimate per SD [95% CI] | p-value |
| **HbA_1c_** | RMSSD | −0.03 [−0.11; 0.06] | 0.52 | −0.03 [−0.12; 0.07] | 0.60 |
|  | LF/HF ratio | −0.10 [−0.20; −0.00] | 0.041 | −0.04 [−0.15; 0.07] | 0.51 |
|  | SDNN | −0.25 [−0.34; −0.17] | <0.0001 | −0.19 [−0.28; −0.09] | 0.00011 |
| **HOMA-IR** | RMSSD | −0.07 [−0.14; 0.01] | 0.087 | −0.08 [−0.17; 0.01] | 0.074 |
|  | LF/HF ratio | −0.07 [−0.16; 0.01] | 0.094 | −0.00 [−0.10; 0.10] | 0.96 |
|  | SDNN | −0.17 [−0.25; −0.09] | <0.0001 | −0.11 [−0.20; −0.02] | 0.016 |
| **C-peptide** | RMSSD | 0.01 [−0.07; 0.09] | 0.79 | 0.01 [−0.08; 0.10] | 0.87 |
|  | LF/HF ratio | −0.14 [−0.22; −0.06] | 0.00088 | −0.06 [−0.16; 0.04] | 0.22 |
|  | SDNN | −0.17 [−0.24; −0.09] | <0.0001 | −0.10 [−0.19; −0.01] | 0.027 |

The $\hat{\beta}$-estimates [per SD] presented are from multivariate regression analyses with HbA_1c_, HOMA-IR, and C-peptide as independent variables and heart rate variability variables as dependent variable. SD, standard deviation; CI, confidence interval; RMSSD, root mean square of successive differences; LF/HF, low-frequency to high-frequency ratio; SDNN, standard deviation of NN intervals.
